# Sudden Death without a Clear Cause after Comprehensive Investigation: An Example of Forensic Approach to Atypical/Uncertain Findings

**DOI:** 10.3390/diagnostics11050886

**Published:** 2021-05-17

**Authors:** Simone Grassi, Mònica Coll Vidal, Oscar Campuzano, Vincenzo Arena, Alessandro Alfonsetti, Sabina Strano Rossi, Francesca Scarnicci, Anna Iglesias, Ramon Brugada, Antonio Oliva

**Affiliations:** 1 Section of Legal Medicine, Department of Health Surveillance and Bioethics, Catholic University of the Sacred Heart, 00168 Rome, Italy; alessandroalfonsetti@outlook.com (A.A.); sabina.stranorossi@unicatt.it (S.S.R.); francesca.scarnicci@unicatt.it (F.S.); antonio.oliva@unicatt.it (A.O.); 2 Cardiovascular Genetics Center, University of Girona-IDIBGI, 17290 Salt, Girona, Spain; mcoll@gencardio.com (M.C.V.); oscar@brugada.org (O.C.); annai@brugada.org (A.I.); ramon@brugada.org (R.B.); 3 Institute of Anatomical Pathology, Department of Woman and Child Health and Public Health, Catholic University of the Sacred Heart, 00168 Rome, Italy; vincenzo.arena@policlinicogemelli.it

**Keywords:** Brugada syndrome, *TTN* gene, *SLMAP* gene, fatty infiltration right ventricle, sino-atrial node fibrosis, myocardial bridging, sudden death, cannabis, forensic pathology, molecular autopsy

## Abstract

Sudden death (SD) is defined as the unexpected natural death occurred within an hour after the onset of symptoms or from the last moment the subject has been seen in a healthy condition. Brugada syndrome (BrS) is one of the most remarkable cardiac causes of SD among young people. We report the case of a 20-year-old man who suddenly died after reportedly having smoked cannabis. Autopsy, toxicology, and genetic testing were performed. Autopsy found a long and thick myocardial bridging (MB) at 2 cm from the beginning of the left anterior descending coronary artery. Furthermore, at the histopathological examination, fibrosis and disarray in myocardial area above the MB, fatty tissue in the right ventricle and fibrosis of the sino-atrial node area were found. Toxicology testing was inconclusive, while genetic testing found a rare missense variant of the *TTN* gene, classified as likely benign, and a variant of unknown significance in the *SLMAP* gene (a gene that can be associated with BrS). Hence, despite several atypical features were found, no inference on the cause of the death could be made under current evidence.

## 1. Introduction

Sudden death (SD) can be defined as the unexpected natural death of a healthy individual occurring within the first hour after the onset of symptoms or, if death is unwitnessed, within 24 h of the victim being seen in a healthy state. Albeit SD can have many causes, it is normally of cardiac origin (sudden cardiac death—SCD), and in those younger than 35 years, it is mainly due to cardiomyopathies and channelopathies [1,2,3]. Brugada syndrome (BrS) is the second most common channelopathy after Long QT syndrome. Sudden death is often the first clinical manifestation of BrS, and this syndrome is not associated with macroscopic/microscopic anomalies, albeit recurrent flogosis and fibrosis in some cardiac districts (e.g., right ventricle outflow tract) have been reported [4,5]. BrS is characterized by autosomal dominant pattern of inheritance, incomplete penetrance and variable expressivity [6]. *SCN5A*, which encodes the pore-forming ion-conducting α-subunit of the cardiac sodium channel (Nav1.5), is the most frequently mutated gene in BrS cases [7]. In particular, the level of expression of Nav1.5 sodium channels is a critical determinant of the arrhythmogenic process, and its decrease may be a sign of BrS in heterozygous patients [8]. In particular, *SCN5A* variants can cause late Na^+^ current and thus the clinical manifestations of both BrS and Long QT syndrome [4,9]. The only diagnostic ECG pattern of BrS (type 1) can occur either spontaneously or in response to many licit (e.g., flecainide) and illicit (e.g., cocaine) drugs [6,10]. These substances should be avoided by carriers of pathogenic variants since this pattern increases the risk of adverse cardiac events (ventricular tachyarrhythmias, cardiac arrest and sudden cardiac death) [10]. Despite there being not strong evidence of an association between cannabis use and type 1 Brugada ECG pattern, a potential favoring role of this substance has been described [11].

In this paper, we report a case of sudden death in a young consumer of cannabis in which the autopsy revealed a long and thick myocardial bridging (MB, i.e., a congenital anomaly represented by an epicardial coronary artery with an intramuscular course), adipositas cordis, fibrosis of the sino-atrial node, and postmortem genetic testing found a variant previously associated to BrS. The aim of the report of this highly challenging case is to depict how sudden unexplained deaths can sometimes be associated to many atypical and complex findings, albeit of unknown significance.

## 2. Case Report

**Figure 1:**
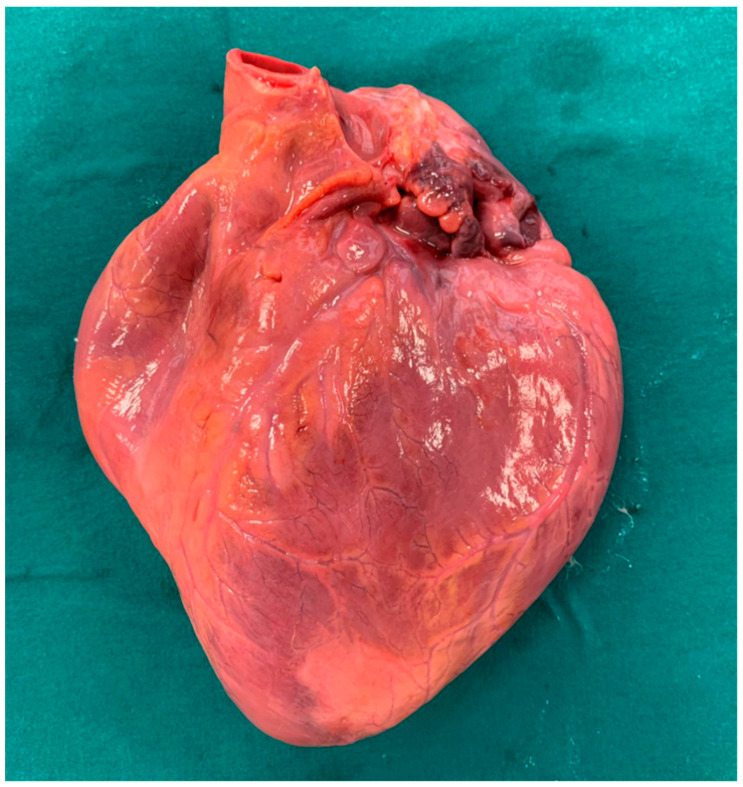
Heart before fixation.

**Figure 2:**
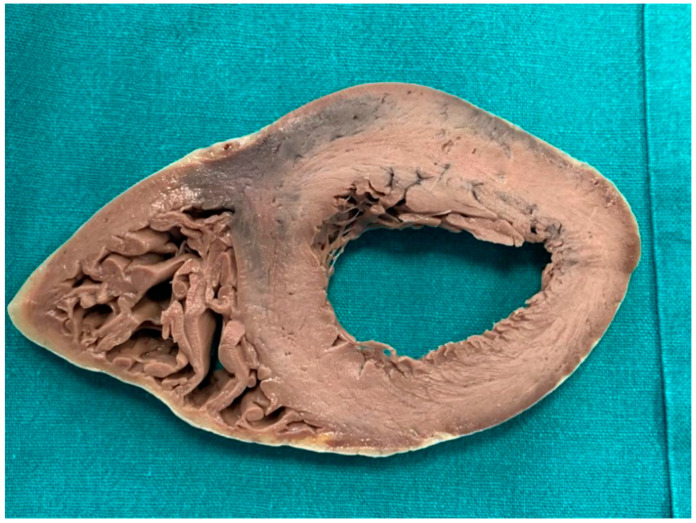
Section of heart after fixation.

**Figure 3:**
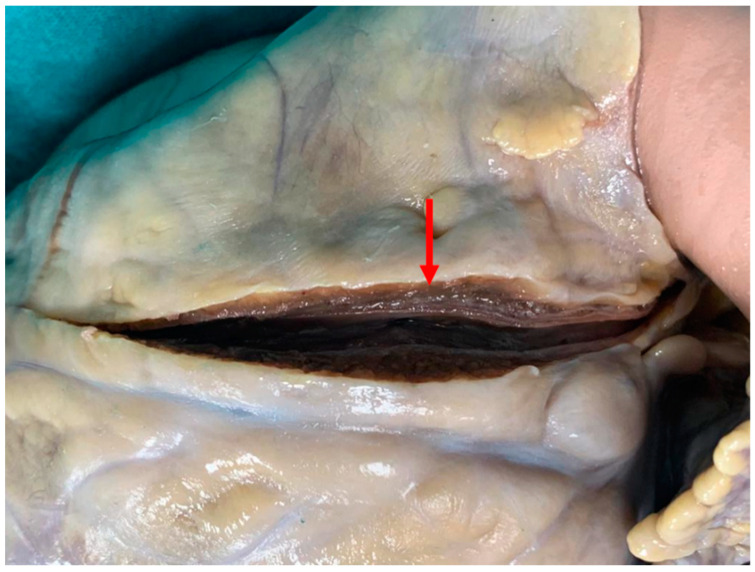
Heart after fixation, myocardial bridging: the left anterior descending coronary artery starts with a normal course, but at 2 cm from its beginning, it tunnels through the myocardium (the 0.3 cm thick, 5.5 cm long tunneled tract is indicated by the red arrow: the dark overlying myocardium bridge is clearly visible).

**Figure 4:**
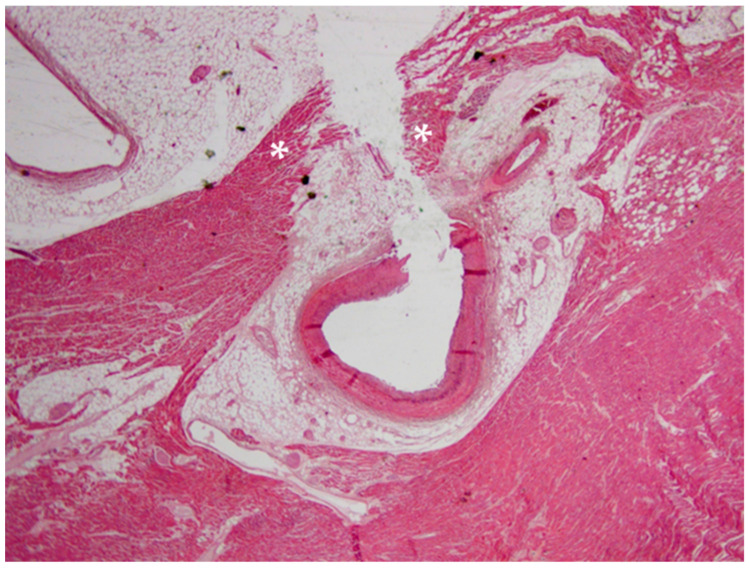
Histopathological image of myocardial bridging: the two parts of the myocardial bridge (disrupted by the coronary dissection) are indicated by white asterisks (Hematoxylin and eosin stain, 10× magnification).

**Figure 5:**
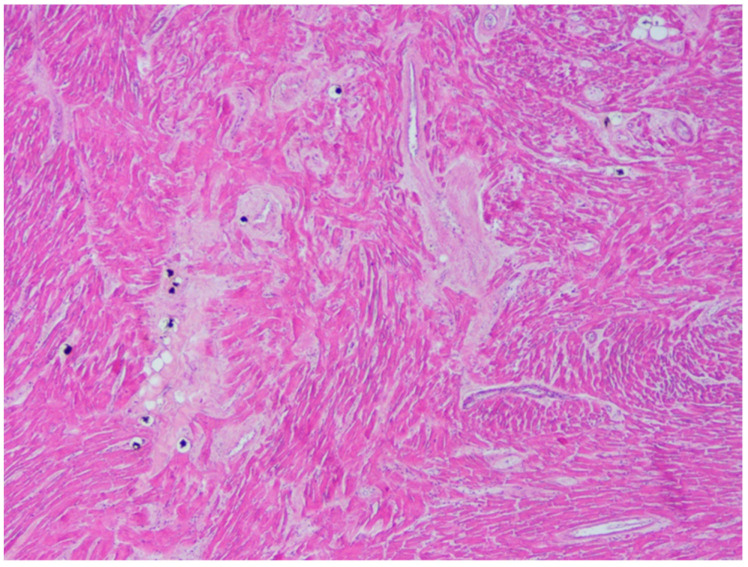
Wavering of myocardial fibers, fibrosis and disarray in the left ventricle myocardial area above the myocardial bridging (Hematoxylin and eosin stain, 200× magnification).

**Figure 6:**
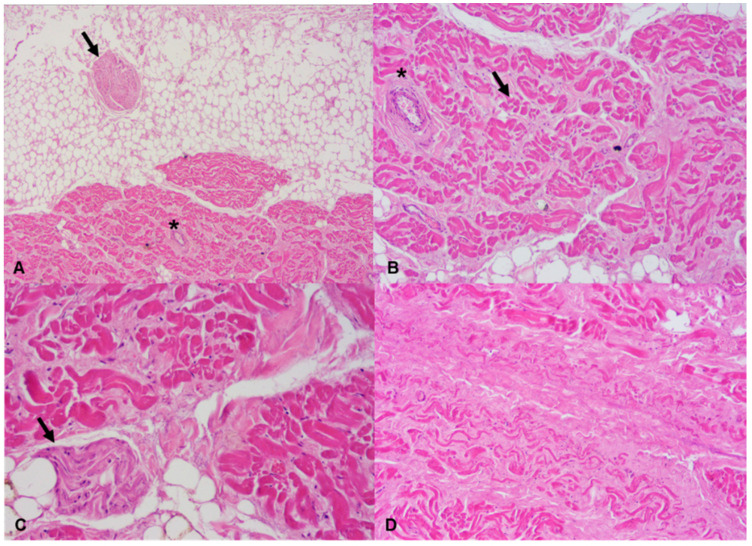
Sino-atrial node: (**A**) sections of a nerve fiber (arrow) near the sino-atrial node and of a branch of the artery of the sino-atrial node (asterisk) (Hematoxylin and eosin stain, 40× magnification); (**B**) section of a branch of the artery of the sino-atrial node (asterisk). A group of pacemaker cells is indicated by an arrow (Hematoxylin and eosin stain, 100× magnification); (**C**) section of a nerve fiber (arrow) running into the sino-atrial node (Hematoxylin and eosin stain, 200× magnification); (**D**) fibrosis of the sino-atrial node can be clearly seen (Hematoxylin and eosin stain, 100× magnification).

**Figure 7:**
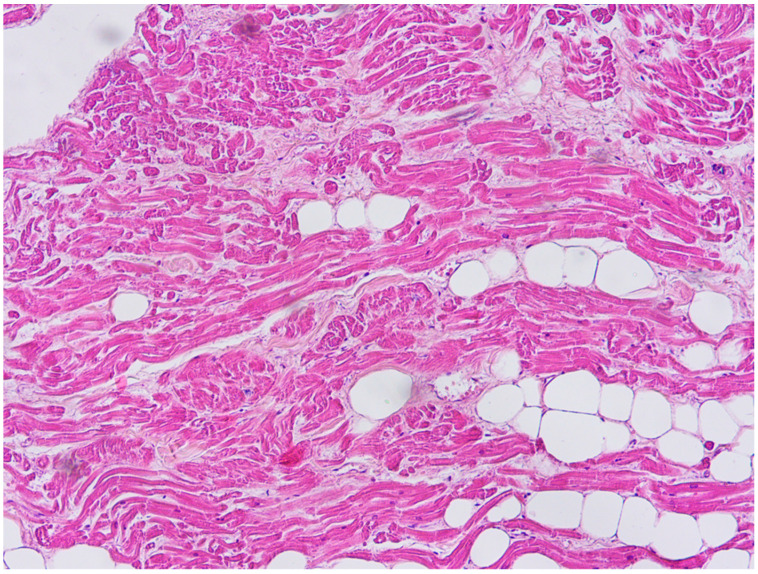
Fatty tissue inside the myocardium in the antero-lateral region of right ventricle free wall (Hematoxylin and eosin stain, 400× magnification).

A 20-year-old young man (body mass index: 28) suddenly died in a public place. The friend who witnessed his death declared that he had recently smoked cannabis. All the witnesses said that the young man appeared in good health and that he suddenly collapsed (without any previous symptom). Basic life support maneuvers were promptly started, but when paramedics arrived, he was asystolic at ECG. The public prosecutor requested an autopsy to find the cause of the death. The victim played several competitive sports and had a syncope while playing football three years before. At external examination of the body no relevant sign was found. At the autopsy, performed five days after the death, the heart was isolated and fixated in toto in a 10% buffered formalin-based solution (Figure 1 and Figure 2). Both the lungs appeared swollen (weights: left—450 g, right—940 g), and multiorgan congestion was observed. Heart examination (weight: 530 g, longitudinal diameter: 11 cm, transverse diameter: 13 cm) presented no anomaly. Atria, valves, left ventricle (anterior wall thickness: 1.3 cm; lateral wall thickness: 1.4 cm; posterior wall thickness: 1.7 cm; interventricular septum thickness: 1.5 cm) and right ventricle (wall thickness: 0.5 cm) did not show any macroscopic relevant finding. Coronary circulation was right dominant. Left anterior descending coronary artery presented, at 2 cm from its beginning, a 0.3-cm-thick 5.5-cm-long myocardial bridging (Figure 3). Other coronary arteries were macroscopically normal. The conduction system was carefully analyzed, and serial sectioning targeted blocks of areas of interest [12,13]. Histopathological examination of the myocardium (Figure 4) found wavering of myocardial fibers, fibrosis and disarray in the left ventricle myocardial area above the MB (Figure 5); fibrosis in sino-atrial node area (Figure 6) and infiltration of fatty tissue (separated the myocardium) in the antero-lateral region of right ventricle free wall (Figure 7). The atrio-ventricular node presented no microscopic anomalies. No signs of myocarditis were found.

## 3. Toxicology Testing

According to the information collected by the police, the victim was a habitual consumer of cannabis. Toxicology testing was performed on peripheral blood and bile. Bladder contained no urine. Traces of THC (tetrahydrocannabinol, i.e., the main psychoactive constituent of cannabis) and a THC-COOH (11-Nor-9-carboxy-Δ9-tetrahydrocannabinol, i.e., the principal secondary metabolite of THC) concentration of 3 ng/mL were found in blood, while a THC concentration of 9 ng/mL and a THC-COOH concentration of 1800 ng/mL were found in the bile.

## 4. Genetic Testing

Postmortem whole blood of the victim underwent extraction with Chemagic MSM I (PerkinElmer, Waltham, MA, USA). Spectrophotometric analysis was performed to evaluate quality ratios of absorbance (260/280:260/230 minimum of 1.8:2.2). DNA concentration was determined using the Qubit fluorometer (Thermo Fisher Scientific, Waltham, MA, USA), and 3 μg of DNA was used for library preparation. NGS analysis was performed using a custom resequencing panel of 82 genes associated with cardiomyopathies and channelopathies, designed and optimized by our own group of bioinformatics (Table 1). The final size was 477.28Kbp (hg19) of encoding regions and UTR boundaries. We sequenced an isoform for each gene and only coding exons and 10 base pairs inside intronic regions. Exons were obtained from Ensembl site version 81 in GRCh38 and translated to hg19 [14]. Bait design was performed through an in-house algorithm and submitted to Agilent SureSelect Design Web. For the chip design, variable tailing bait and variable multiplicity bait were used to obtain homogeneous coverage and to optimize sample load. Genomic DNA was fragmented through sonication using the Bioruptor (Diagenode, Denville, NJ, US). The 82 genes were enriched using the SureSelect Custom Target Enrichment System Kit (Agilent Technologies, Santa Clara, CA, USA) following the manufacturer’s instructions for the “SureSelect Target Enrichment System for Illumina Paired-End Sequencing version B.1” (SureSelect XT Custom library, Agilent Technologies, Santa Clara, CA, USA). The paired-end sequencing process was carried out on MiSeq System (Illumina, Inc., San Diego, CA, USA) using 2 × 76 bp read length.

NGS analysis was submitted to an in-home pipeline [15]. Variant calls for SNVs and Small Indels obtained using Samtools (v.1.3.1) and internal Gendicall Caller. Population data were obtained from dbSNP [16], 1000 Genomes Project [17], Exome Variants Server (EVS) (Exome Variant Server, NHLBI GO Exome Sequencing Project (ESP), Seattle, WA, 2014), Exome Aggregation Consortium (ExAC) Cambridge, MA [18], and Genome Aggregation Database (gnomAD; http://gnomad.broadinstitute.org, accessed on 13 May 2021). The protein predictors consulted were PolyPhen2 [19], Sift [20], Provean [21] and Mutation Taster [22]. Finally, we also used the splicing predictors MaxEntScan [23], FSPLICE, GeneSplicer [24] and NNsplice [25]. Sanger sequencing was indicated when the coverage was lower than 30X, as well as to validate variants with a frequency lower than 1%. Hence, polymerase chain reaction (PCR) was performed, and after a purification through ExoSAP-IT (USB Corporation, Cleveland, OH, USA) the product was directly sequenced using the dideoxy chain-termination method in an ABI Prism Big Dye^®^ Terminator v3.1 Cycle Sequencing Kit (Applied Biosystems, Waltham, MA, USA). Sequencing was analyzed using a 3130XL Genetic Analyzer (Applied Biosystems, Waltham, MA, USA) and SeqScape Software v2.5 (Life Technologies, Waltham, MA, USA). Genetic variants were reported in compliance with the recommendations given by the Human Genome Variation Society (HGVS). As already reported, samples were re-analyzed when similar levels of coverage between samples were found [26]. Variants were classified following current recommendations [27,28,29].

## 5. Genetic Results

The average coverage of the massive sequencing in this sample was 183 and the call rate at 30× was 99.835%. Excluding the synonymous variants, we identified two rare missense variants: c.599C > T_SLMAP and c.76006A > G_TTN. The variant of *SLMAP* leads to a change of a serine (Ser) to a leucine (Leu) in the p.200 position. The variant of *TTN* causes a change of threonine (Thr) to alanine (Ala) in the p.25336 position. These variants had been previously identified, and the corresponding code was rs1474269656 (*SLMAP*) and rs1320086002 (*TTN*). They are not present in the Human Gene Mutation Database (HGMD). The minor allele frequency was consulted in Gnomad database: the allelic frequency of the variant in *SLMAP* gene was 0.0003984%, while the allelic frequency of the variant of the *TTN* gene was 0.0004032%. About the in silico tools predictors, in the case of the *SLMAP* variant, all the tools predicted a pathogenic role, whereas in the case of the TTN variant, only one out of three predicted pathogenicity (Table 2).

Applying current criteria for the assessment of the significance of the variants [27], the significance of variant of *TTN* gene was interpreted as likely benign, while the variant of *SLMAP* was classified as of unknown significance. We collected and analyzed blood samples from the relatives of the victim to study the segregation of this variant and thus to evaluate if in other members of the family it was associate to a particular phenotype. The analysis found that the variant was transmitted by his mother. Both the relatives reported no previous cases of SD or cases of arrhythmogenic syndromes in their families, were visited by a cardiologist and underwent resting 12-lead ECG and echocardiography, which were negative. In the light of this data, we confirmed the unknown significance of the variant.

## 6. Discussion

Sudden unexplained death cases are often thought to be completely “silent” at the autopsy. On the contrary, in our case we found several atypical (and common) features (myocardial bridging surrounded by signs of hypertrophic cardiomyopathy (HCM), fatty infiltration of the right ventricle, significant fibrosis of the sino-atrial node, variant-of unknown significance of a gene associated with Brugada syndrome and cannabis consumption before the SD), but none of them is of clear clinical significance.

### 6.1. Autopsy Findings

At the autopsy, the only relevant macroscopic finding was the myocardial bridging of left anterior descending coronary artery in the context of a normal heart. In general terms, MB is a congenital coronary anomaly (mean length: 14.64 ± 9.03 mm, mean thickness: 1.23 ± 1.32 mm) that usually affects (as in our case) the middle tract of left anterior descending coronary artery and is often found for the first time only at autopsy [30,31,32]. According to many authors, when it is particularly thick (2 mm is generally indicated as cut-off), it may cause myocardial ischemia, but it is controversial if it can be considered a possible cause of SCD [32,33]. Myocardial bridging has been also reported in men with no family history of sudden cardiac death in which the first clinical manifestations of BrS were acute chest pain and syncope [34,35]. A causal relationship between this coronary anomaly and BrS has never been proved, but Riezebos et al. hypothesized that “interaction between the provoked ischemia and the specific repolarization abnormalities that are caused by the Brugada syndrome could provide an electrophysiological substrate that may increase individual susceptibility to life-threatening ventricular tachyarrhythmias” [35].

Microscopic examination of the atria and ventricles found some interesting and complex features. Starting from the left ventricle, fibrosis and disarray in the myocardial area above the MB were described. These features have already been associated to MB in young cases of SD [32,36]. In particular, considering the young age, the presence of fibrosis and disarray without ventricular hypertrophy in regions far from the septum (as in our case) is compatible with an early phenotype of HCM (but it has also been reported as a sign of MB-related myocardial ischemia) [32,36,37]. It should be considered that the abnormal (>500 g) weight of the heart is also suggestive of HCM.

In our case, there also was a sign suggestive of arrhythmogenic cardiomyopathy (ACM): the fatty infiltration of the right ventricle. However, this feature was only found in the antero-lateral region of the right ventricle, a localization that is typical of physiological conditions [38]. Moreover, the fatty infiltration was clearly separated by the myocardium, and there were no signs of necrosis/atrophy of the myocytes (that are typical of ACM), no fibrous or fibro-fatty infiltration of the myocardium (common among young cases of ACM) and no inflammatory infiltrates (that can be present, for example, in ACM hot phases, i.e., the phases of acute myocarditis that can suddenly complicate the disease) [37,39]. Finally, NGS found no variant pathogenic for ACM. All these data suggest a physiological condition. However, this finding is atypical, because fatty infiltration of the myocardium generally affects elderly or obese patients, and our case was young and non-obese (albeit overweight). Hence, since the absence of signs of cardiomyopathy, a clear pathogenic significance cannot be given to the findings. However, it should be considered that some authors reported that fatty infiltration could cause arrhythmias because it represents a structural barrier to the heart electrical impulse and causes oxidative stress. In particular, previous cases of sudden death in patients with fat of the free wall of the right ventricle have been reported [40].

Another interesting microscopic feature was the fibrosis of the sino-atrial node area. In sudden death cases, it is important that an expert pathologist carefully examines the conduction system, as recommended, among others, by Ottaviani and Buja and by The Royal College of Pathologists [12,13]. Indeed, this analysis can help to find the cause of the death but is complicated, for example, by the interindividual anatomical variability [12]. The feature we found in our case is considered physiological (to at least some extent) since fibrosis has been described as a “modulator of structural and functional integrity” of sino-atrial node [41]. In our case, the found amount of fibrosis in the sino-atrial node area is relatively unusual for the young age of the victim (fibrosis is generally more extended in older patients) [41]. However, despite the crucial function of this pacemaker and the presence of important structures, such as Nav1.5 channels-in the periphery of its area, according to current evidence, no pathological significance of this feature can be assessed [41,42].

In conclusion, the autopsy revealed a long and thick MB located in a very proximal tract of the coronary artery and surrounded by signs suggestive of HCM/MB-caused ischemic damage. Moreover, fatty infiltration of the right ventricle and significant fibrosis of the sino-atrial node area were found. These features are relatively common in the general population but are atypical in a young and healthy patient.

### 6.2. Toxicology Findings

In our case, the victim was reportedly a habitual cannabis consumer, and the friend who witnessed his death told the police that he had smoked cannabis few tens of minutes before his death. Toxicologic testing was positive for cannabis use. However, THC concentration in peripheral blood was low. Marijuana use has been associated with a wide spectrum of cardiac adverse effects and, in particular, with arrhythmias (e.g., atrial fibrillation/flutter, atrioventricular block/asystole, sick sinus syndrome and ventricular tachycardia) [11]. However, in our case, the low level of blood THC and the absence of urine do not allow to make inference on the time of the assumption.

### 6.3. Molecular Autopsy Findings

Currently, postmortem genetic testing is not mandatory, but in the cases of suddenly died young persons it can often give a substantial contribution to find the cause of the death [32,43,44,45,46]. In general terms, the diagnostic yield of molecular autopsy performed through Next-Generation Sequencing mainly depends on the chosen approach (target sequencing vs. whole exome sequencing vs. whole genome sequencing), on the specific arrhythmogenic syndrome (it varies from 15–30% for Short QT syndrome to 80–85% for Long QT syndrome) and on the presence of other cases of disease in the family (it is generally higher in familial rather than in sporadic cases) [32,44]. In Brugada syndrome, the diagnostic yield is 30–35% [32,44].

In our case, we identified two rare missense variants in the index case, affecting *TTN* and *SLMAP*. Both of them can be considered variants rather than polymorphisms since they are associated to a very low frequency in the general population. Since the sequencing was not genome-wide, the presence of variants in genes not included in the panel could not be excluded.

*TTN* gene codifies for a very large protein called titin, that an important role in skeletal muscles and myocardium contractile functions. Mutations in this gene have been related to familial dilated cardiomyopathy, arrhythmogenic right ventricular cardiomyopathy or familial HCM. However, the variant we found had previously been described and was classified as likely benign. Anyway, since *TTN* gene has been associated with HCM before, and in our case, some signs of HCM were found, and experimental studies should be undertaken to certainly assess the significance of this variant (although two in silico tools suggest it is likely benign).

The variant c.599C > T of the *SLMAP* gene is a very interesting finding because sarcolemmal membrane-associated protein gene has previously been associated with Brugada syndrome [47,48]. As said, the significance of this variant is still unknown, but all the three in silico tools predictors consulted suggest its pathogenicity.

Hence, we analyzed scientific literature on this theme to verify if cannabis smoking and Brugada syndrome can have combined pro-arrhythmogenic effects.

### 6.4. Association between Brugada Syndrome and Cannabis

Relatively few associations between Brugada syndrome and cannabis have been reported so far. Daccarrett et al. described the case of a 19-year-old young man who showed Brugada-like ST segment elevation in V1 and V2 few minutes after cannabis consumption [49]. Romero et al. presented the case of a 42-year-old habitual cannabis consumer who manifested type I Brugada pattern and frequent premature ventricular beats at the ECG after smoking cannabis [50]. A similar case was reported by Doctorian and Chou: a 43-year-old man who, after smoking cannabis, experienced ventricular fibrillation twice in six months and was then diagnosed with type-1 Brugada syndrome [51]. It is interesting that the first ventricular fibrillation arrest happened two hours after smoking cannabis, hence after a relevant amount of time. Finally, Kariyanna presented the case of a 38-year-old showing an ECG Brugada type-1 pattern after having frequently smoked cannabis in the last 30 h [52]. However, at the best of our knowledge, in scientific literature, a forensic case of SD in the young after smoking cannabis with genetic variants suggestive of Brugada syndrome has never been reported. Few cases of SUD after cannabis consumption have been reported: for example, Hartung et al. performed molecular autopsies in two cases of this kind, but they did not find any variant (only polymorphisms) [53]. In the light of these data, an association between acute and/or chronic consumption of cannabis and BrS-related cardiac events is theoretically plausible but still uncertain. Hence, in our case, if the failure to establish the time of consumption is also considered, the cannabis use cannot be indicated as the cause of the death.

## 7. Conclusions

In our case, several findings of uncertain significance were found at the autopsy and the postmortem genetic testing. In particular, the thick and long myocardial bridging surrounded by microscopic changes of the ventricular wall suggestive of HCM, the presence of significant fibrosis of the sino-atrial node and fatty infiltration of the right ventricle in a healthy and young patient and the variant of the *SLMAP* gene could lead to hypothesize an arrhythmic death, but these features must be considered of uncertain significance according to current evidence. Since there was no urine in the bladder, the results of toxicological testing cannot help to individuate the time of the cannabis assumption, and thus, a cannabis-induced arrhythmia cannot be hypothesized. The characteristics (length, thickness, location) of the MB and the in silico predictions regarding *SLMAP* variant suggest that these findings could have caused a fatal arrhythmia, but nothing can be stated under current evidence. In our opinion, this case depicts the complexity of forensic investigation in sudden unexplained deaths and shows as sudden unexplained deaths can sometimes be anything but silent. Sharing data regarding findings in sudden unexplained deaths, even when a cause of death is not found, is crucial to help to interpret common findings like MB and genetic variants of unknown significance.

## Figures and Tables

**Table 1:** List of the genes included in the custom resequencing panel.

Genes Included in the Custom Resequencing Panel
*ABCC9, ACTC1, ACTN2, AKAP9, ANK2, BAG3, CACNA1C, CACNA2D1, CACNB2, CASQ2, CAV3, CRYAB, CSRP3, DES, DMD, DMPK, DSC2, DSG2, DSP, EMD, FKTN, FLNC, GLA, GPD1L, HCN4, JPH2, JUP, KCND3, KCNE1, KCNE2, KCNE3, KCNE5, KCNH2, KCNJ2, KCNJ5, KCNJ8, KCNQ1, LAMP2, LDB3, LMNA, MYBPC3, MYH6, MYH7, MYL2, MYL3, MYOZ2, MYPN, NEBL, NEXN, NOS1AP, PDLIM3, PKP2, PLN, PRKAG2, RANGRF, RBM20, RYR2, SCN1B, SCN2B, SCN3B, SCN4B, SCN5A, SCN10A, SGCD, SLMAP, SNTA1, TAZ, TCAP, TGFB3, TMEM43, TMPO, TNNC1, TNNI3, TNNT2, TP63, TPM1, TRDN, TRIM63, TRPM4, TTN, TTR, VCL*

**Table 2:** Pathogenicity predictions according to different in silico tools.

Variant	Polyphen2	Provean	Mutation Taster
c.599C > T_SLMAP	Possibly damaging	Deleterious	Disease causing
c.76006A > G_TTN	Benign	Deleterious	Polymorphism

## Data Availability

Not applicable.
